# 

**Published:** 1856-02

**Authors:** 


ARTICLE I.—An Essay on Pneumonia, read before the Atlanta Medical Society. By THOS. M. DARNALL, M. D., Dec. 6, 1855 .......... 321

ARTICLE II.—Report of a case of Syphilis, with Remarks. By W. F. WESTMORELAND, M. D., Professor of Surgery in the Atlanta Medical College.... 327

ARTICLE III.—Report of Medical Clinic, continued. By J. G. WESTMORELAND, M. D., Professor of Materia Medica, &c., in the Atlanta Medical College .......... 329

ARTICLE IV.—Thesis on the Signs of Pregnancy, presented to the Faculty of the Atlanta Medical College. By J. JUNIUS NEWSOME, of Sandersville, Georgia, 1855 .......... 333

### *SELECTIONS.*

Malaria not the Cause of Fever .......... 342

Abortion following the Administration of Chloroform .......... 348

Clinical Lecture at St. John's Hotel for Invalids .......... 351

Fibrinous Bodies in the Heart after Death .......... 355

Report of the Committee on Scarlatina .......... 360

Semile Insanity .......... 363

Editorial and Miscellaneous .......... 306

